# A *de novo* heterozygous *PSTPIP1* variant associated with PAPA syndrome: a Chinese case report and literature review

**DOI:** 10.3389/fgene.2026.1825761

**Published:** 2026-06-11

**Authors:** Mengmeng Wang, Ping Zhang, Min Shen

**Affiliations:** 1 Department of Rare Diseases, Peking Union Medical College Hospital (PUMCH), Chinese Academy of Medical Sciences and Peking Union Medical College; State Key Laboratory of Complex Severe and Rare Diseases, PUMCH, Beijing, China; 2 Department of Rheumatology and Immunology, Tianjin First Central Hospital, Tianjin, China

**Keywords:** autoinflammatory diseases, PSTPIP1-associated inflammatory disease, PAPA syndrome, *PSTPIP1* gene, TNF-α inhibitor

## Abstract

Pyogenic arthritis, pyoderma gangrenosum, and acne (PAPA) syndrome is a rare autosomal dominant hereditary autoinflammatory disease caused by *PSTPIP1* gene variants and belongs to the *PSTPIP1*-associated inflammatory diseases (PAIDs). Its core clinical manifestations include recurrent pyogenic arthritis, pyoderma gangrenosum, and severe acne with onset in childhood or adolescence. Some patients may also present with multisystem involvement, such as inflammatory bowel disease and scleritis. Inflammation markers, such as CRP and ESR, are often significantly elevated. Treatment mainly involves targeted inhibition of inflammatory pathways, such as IL-1 inhibitors and TNF-α inhibitors. In this article, we report a Chinese patient with PAPA syndrome with disease onset at 13 years of age, whose main manifestations were pyoderma gangrenosum and acne. Genetic testing revealed a *de novo PSTPIP1* gene variant (c.748G>A, p.Glu250Lys). We also reviewed recent literature on PAPA syndrome, summarizing its clinical manifestations, diagnosis, and treatment to enhance physicians’ understanding of the condition.

## Introduction

Pyogenic arthritis, pyoderma gangrenosum, and acne (PAPA) syndrome is a rare autosomal dominant autoinflammatory disorder belonging to the *PSTPIP1*-associated inflammatory disease (PAID) spectrum, driven by gain-of-function mutations in the *PSTPIP1* gene encoding proline–serine–threonine phosphatase-interacting protein 1. *PSTPIP1* mutations disrupt the protein–protein interactions between PSTPIP1 and pyrin, leading to excessive activation of the inflammasome, overproduction of pro-inflammatory cytokines, such as interleukin-1β and tumor necrosis factor-α, and sustained sterile inflammation, which constitutes the core pathogenic mechanism of all PAID disorders. This rare monogenic disease is characterized by recurrent sterile inflammatory episodes affecting the joints, skin, and sebaceous glands, manifesting as destructive arthritis, painful ulcerative pyoderma gangrenosum, and severe cystic acne, which severely impairs patients’ quality of life and long-term prognosis. Lindor et al. (1997) reported the first case in 1997, and as of 2020, a total of 87 cases had been publicly reported worldwide in the literature. Since 2020, approximately 15 cases of PAPA syndrome have been publicly reported worldwide, among which 6 cases were reported in China (Wang et al., 2021). The global prevalence of PAPA syndrome remains poorly defined, with fewer than 100 confirmed cases reported worldwide to date, predominantly identified in Caucasian populations and extremely rare in East Asian cohorts. Its clinical presentation exhibits marked heterogeneity across age groups: pediatric patients primarily present with recurrent sterile pyogenic arthritis, which may progress to joint destruction and disability if untreated; adolescents and young adults typically develop severe cystic acne and pyoderma gangrenosum, while some atypical patients display overlapping phenotypes with other autoinflammatory diseases, including PAPA-like syndromes characterized by milder inflammatory manifestations or the incomplete disease triad (Hajialigol et al., 2023). The broad PAID spectrum also encompasses PASH (pyoderma gangrenosum, acne, and hidradenitis suppurativa) syndrome, PAPASH (pyogenic arthritis, pyoderma gangrenosum, acne, and hidradenitis suppurativa) syndrome, PAMI (PSTPIP1-associated myeloid-related proteinemia inflammatory) syndrome, PASS (pyoderma gangrenosum, acne vulgaris, hidradenitis suppurativa, ankylosing spondylitis) syndrome, PsAPASH (psoriatic arthritis, pyoderma gangrenosum, acne, hidradenitis suppurativa) syndrome, and PAC (pyoderma gangrenosum, acne, ulcerative colitis) syndrome. This article aims to provide a detailed report on the specific case of a newly discovered Chinese patient with PAPA syndrome and take this opportunity to comprehensively review and summarize the molecular pathogenesis, diverse clinical manifestations, key diagnostic criteria, and relevant diseases that need to be differentiated, along with the current mainstream treatment strategies and management plans for this disease.

## Case presentation

A 16-year-old Chinese Han boy reported cutaneous folliculitis-like changes in the lower extremities for 10 years and began to exhibit increasingly noticeable facial acne at 14 years. He was treated with topical ointment, but no improvement was observed. He has presented recurrent scattered indurated rashes on both lower limbs since age 13, with the largest lesion measuring approximately 6 × 8 cm^2^, accompanied by discharge. He was also found to have a low level of white blood cell count of 2–3 × 10^9^/L. Skin lesions usually progressed to ulceration after several days. Incision and drainage, along with anti-infective treatment, were performed, but no improvement was observed, and purulent secretions gradually appeared. Pathological examination of skin biopsy in local hospitals revealed intrapapillary pustules accompanied by neutrophilic spongiform edema, with a few acantholytic cells observed within the pustules. There were hemorrhage and edema in the dermal papilla, with infiltration of neutrophils, eosinophils, and lymphocytes. A diagnosis of PG was considered. Methylprednisolone 40 mg once daily and cyclosporine 75 mg twice daily were administered with unsatisfactory results. Meanwhile, he developed paroxysmal abdominal pain accompanied by mild vomiting and hematochezia, without fever or oral ulcers. Empirical anti-infective treatment was ineffective. After increasing the dosage of methylprednisolone to 120 mg per day, the abdominal pain improved and the skin lesions on the body showed further improvement. Gastrointestinal endoscopy and bone marrow showed no significant abnormalities. Subsequently, adalimumab 40 mg subcutaneously every 2 weeks was given, and the steroid dosage was gradually reduced to prednisone 5 mg per day for maintenance. His symptoms of PG and abdominal pain were relieved, but severe acne did not improve. During the disease course, he had no fever, arthralgia or arthritis.

**FIGURE 1:**
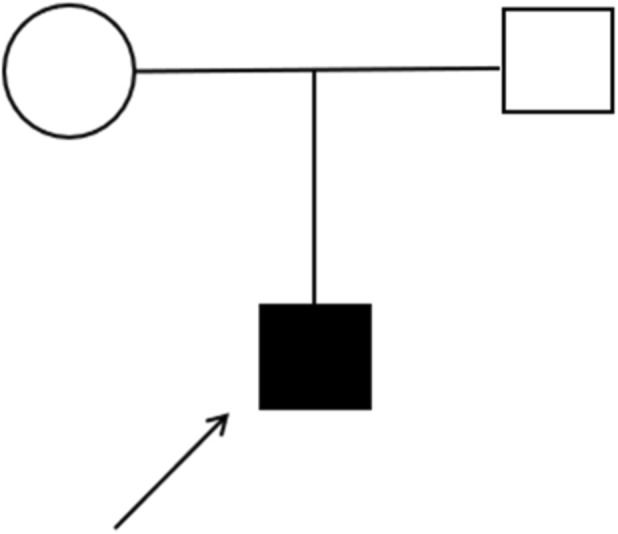
Pedigree of the patient. Arrow, proband; black symbols, affected individuals; open symbols, unaffected individuals.

The patient denied any history of underlying diseases. There was no consanguinity between his parents. They both denied having a history of skin or joint diseases. Furthermore, neither of his parents exhibited any relevant phenotypes similar to those observed in the patient (Figure 1).

**FIGURE 2:**
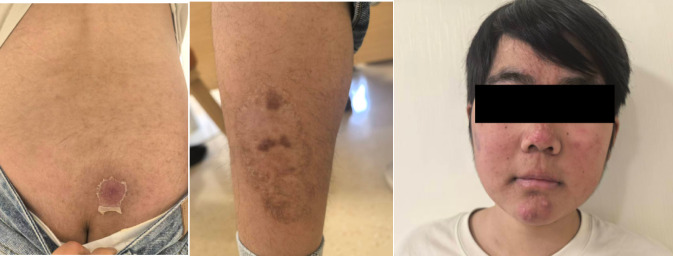
Skin lesions on the sacrococcygeal region, left leg, and face of the patient.

Physical examination revealed that he was a student, was 170 cm tall, and weighed 45 kg. There were multiple red papules and pustules on his face and forehead, with white pus visible on the top of the papules. Some lesions were fused, and some showed pigmentation. Scattered pustule-like lesions were observed on the back, with the largest one located at the sacrococcygeal region, measuring approximately 4 × 3 cm^2^, without ulceration or tenderness. An old scar measuring approximately 10 × 9 cm^2^ was observed on the anterior tibia of the left lower leg (Figure 2). There were no oral ulcers. No superficial lymph node enlargement was palpable. The heart and lungs showed no obvious abnormalities. There was mild tenderness in the right lower abdomen and around the umbilicus and mild hepatosplenomegaly. No abnormalities were found on joint examination.

Complete blood counts showed a white blood cell count of 3.75 × 10^9^/L, a neutrophil count of 1.68 × 10^9^/L, a lymphocyte count of 1.61 × 10^9^/L, a hemoglobin level of 107 g/L, and a platelet count of 199 × 10^9^/L. C-reactive protein (CRP) was 26.39 mg/L, and the erythrocyte sedimentation rate (ESR) was 50 mm/h. Stool occult blood tests were negative. Antinuclear antibodies were negative. Immunoglobulin and complement levels were normal. Plasma calprotectin is 330 ng/mL (normal value <850 ng/mL). Serum zinc level is 12.6 μmol/L (normal range is 10–18 μmol/L). Abdominal ultrasound revealed an oblique diameter of 129 mm in the right hepatic lobe, extending 40 mm below the costal margin, while the spleen was significantly enlarged, with an intercostal thickness of 63 mm and extension 30 mm below the costal margin. Gastrointestinal endoscopy showed no significant abnormalities. Trio whole-genome sequencing revealed a *de novo* heterozygous *PSTPIP1* gene variant (NM_003978.5): exon 11: c.748G>A (p.Glu250Lys). The evidence for pathogenicity includes PM5+PS4+PM2_Supporting+PS2. According to the guidelines of the American College of Medical Genetics and Genomics (ACMG) (Richards et al., 2015), it is determined as a pathogenic variation.

PAPA syndrome was confirmed. The patient was advised to use IL-1 antagonists for better control of the symptoms. Since the disease follows an autosomal dominant inheritance pattern, prenatal gene screening was recommended.

## Discussion

The pathogenesis of PAPA syndrome is closely associated with variants in the *PSTPIP1* gene (proline–serine–threonine phosphatase-interacting protein 1) located on chromosome 15. The *PSTPIP1* gene encodes the PSTPIP1 protein, which consists of 418 amino acids (David et al., 2016). The N-terminal of the PSTPIP1 protein contains a PYD (pyrin domain) domain, capable of interacting with PYD domain proteins such as NLRP3 (NOD-like receptor family pyrin domain containing 3) and ASC (apoptosis-associated speck-like protein containing a CARD) (Hogquist et al., 2023). Disease-associated variants of PSTPIP1 enhance its binding affinity to pyrin, leading to overactivation of the pyrin inflammasome. Once activated, the inflammasome promotes the activation of caspase-1, which then cleaves the precursors of pro-inflammatory cytokines such as interleukin-1β (IL-1β) and interleukin-18 (IL-18) (Petty et al., 2021), converting them into their active forms and releasing them outside the cell. IL-1β is a potent pro-inflammatory factor that ultimately causes autoinflammation in the body, leading to disease onset.

Arthritis and dermatitis are the main clinical manifestations. Arthritis usually appears before dermatitis (Tan et al., 2022). Pyogenic aseptic arthritis often manifests for the first time during childhood and can occur spontaneously or be induced by minor trauma. The affected joints are primarily large joints, with a few cases involving small joints of the hands and feet. It typically presents as an acute onset, characterized by joint swelling, pain, and limited mobility. The joint effusion may be purulent or bloody, resembling the manifestations of infectious arthritis, but joint fluid culture is negative. Recurrent episodes of arthritis can lead to joint destruction, ultimately resulting in joint deformities that affect daily activities.

Cystic acne is the most common type of skin damage, often becoming prominent after puberty. Acne is mostly distributed in areas rich in sebaceous glands, such as the face, chest, back, and shoulders, manifesting as multiple nodules and cysts. Some may form sinus tracts, leaving scars or pigmentation after healing. Compared to ordinary acne, acne associated with PAPA syndrome is more severe, recurrent, and poorly responsive to conventional acne treatments such as antibiotics and retinoids (Lee et al., 2024). Another manifestation of skin damage is necrotizing fasciitis, which is relatively rare but more severe than acne. It often affects the skin of the lower limbs and can also occur on the trunk, buttocks, and other areas. The initial stage of the skin lesion presents as red papules and pustules, rapidly progressing to ulcers with unclear borders and uneven bases, covered with yellow–green purulent secretions. The surrounding skin is red, swollen, and significantly painful. After healing, it forms atrophic scars or keloids (Wang and Liu, 2020).

Notably, not all PAPA syndrome patients present with all three symptoms of pyogenic arthritis, PG, and acne simultaneously. Some patients may only exhibit two of these manifestations (Tallon and Corkill, 2006). Currently, there is no unified international diagnostic standard for PAPA syndrome. Diagnosis is primarily based on clinical symptoms, family history, and genetic testing results. The presence of at least two of the following conditions—pyogenic arthritis, PG, and acne, with recurrent episodes—is required. The detection of known pathogenic variants in *PSTPIP1*, such as E250K, A230T, and E250Q, or new potential pathogenic variants (Boursier et al., 2021) through genetic testing, can confirm the diagnosis of PAPA syndrome. For our patient, combined with his manifestations and gene results (E250K), he could be definitely diagnosed as PAPA syndrome, though he had only two symptoms of this disorder.

In addition to joint and skin symptoms, patients with PAPA syndrome may also experience involvement of other systems, albeit with a relatively low incidence. For instance, they may develop ophthalmia, enteritis, and sometimes systemic symptoms such as low-grade fever, fatigue, and weight loss (Guarneri and Ceccherini, 2022). Our patient had previously experienced abdominal pain and gastrointestinal bleeding during the course of the disease. After excluding other diseases and based on his significantly improved symptoms after the use of steroids and adalimumab, it could be inferred that his gastrointestinal symptoms were probably related to PAPA syndrome.

Variants of the *PSTPIP1* gene can not only cause PAPA syndrome but also cause several other diseases, such as PASH syndrome, PAPASH syndrome, PAMI syndrome, PASS syndrome, PsAPASH syndrome, and PAC syndrome. These diseases have overlapping clinical manifestations and share a common genealogy, so they all belong to the PAID spectrum. Table 1 summarizes the clinical manifestations, associated *PSTPIP1* mutations, and affected protein domains of representative PAID syndromes. For a comprehensive classification of all PAID subtypes, readers are referred to the systematic review by Sanz-Cabanillas et al. (2024).

**TABLE 1:** Genetic and core clinical characteristics of representative PAID syndromes.

Syndrome	Core clinical manifestation	Associated *PSTPIP1* mutation
PAPA	Pyogenic arthritis, acne, pyoderma gangrenosum	p.A230T, p.E250Q, p.E250K, p.D246N, p.E256G, p.D266N, p.G258A, c.G904A
PAMI	Pyogenic arthritis, acne, pyoderma gangrenosum, hyperzincemia, hypercalprotectinemia, neutropenia	p.E250K, p.E257K
PAPASH	Pyogenic arthritis, acne, pyoderma gangrenosum, hidradenitis suppurativa	p.E277D
PASH	Acne, pyoderma gangrenosum, hidradenitis suppurativa	p.Y345C, p.A405C, increased CCTG microsatellite repeats
PASS	Acne, pyoderma gangrenosum, hidradenitis suppurativa, ankylosing spondylitis	—
PsAPASH	Pyoderma gangrenosum, acne, hidradenitis suppurativa, psoriatic arthritis	—
PAC	Pyoderma gangrenosum, acne, ulcerative colitis	p.G403R

Data were summarized and rearranged from Sanz-Cabanillas et al. (2024).

Currently, there is no curative treatment for PAPA syndrome. Steroids can be used for short-term application during acute inflammation to avoid the side effects of long-term high-dose application. Biologics have been the mainstay of treatment for PAPA syndrome in recent years, targeting inflammatory factors such as IL-1β and TNF-α. IL-1β antagonists are the first-line biologics for the treatment (Garcia et al., 2024), including anakinra and canakinumab. TNF-α inhibitors such as etanercept, infliximab, and adalimumab can be used for patients who are intolerant to or have failed to respond to IL-1β antagonists, but their efficacy is inferior to that of IL-1β antagonists. As for our patient, although his symptoms were partially relieved after treatment with TNF-α inhibitors, IL-1β antagonists were recommended for better control of symptoms and inflammatory markers.

## Data Availability

The original contributions presented in the study are included in the article/supplementary material, further inquiries can be directed to the corresponding author.
